# Investigating covert awareness in individuals with Alzheimer’s Disease using functional near‐infrared spectroscopy (fNIRS)

**DOI:** 10.1002/alz.087419

**Published:** 2025-01-09

**Authors:** Garima Gupta, Matthew Kolisnyk, Karnig Kazazian, Rafeh Shahid, Diana Urian, Sergio Novi, Koula Pantazopoulos, Androu Abdalmalak, Jonathan D Huntley, Derek Debicki, Stephen H Pasternak, Adrian Owen

**Affiliations:** ^1^ Western University, London, ON Canada; ^2^ Parkwood Institute/St Joseph's Healthcare Center, London, ON Canada; ^3^ University College London, London UK; ^4^ University of Western Ontario, London, ON Canada

## Abstract

**Background:**

Alzheimer’s disease (AD) is a form of dementia that impairs memory, language, and daily functioning. With disease progression, AD patients reportedly experience disturbances in their awareness of self, others, and their environment. These disturbances are associated with unfavourable clinical outcomes, which prompts critical questions about how AD patients experience the world around them. The present study utilizes a validated neuroimaging paradigm to ‘map’ conscious experiences through changes in brain activity. The premise is that similarity (i.e., synchrony) in conscious experiences of different people can be detected by investigating activity in fronto‐parietal areas linked with higher‐order processing essential for plot‐following. This paradigm was previously used to assess conscious states of behaviorally non‐responsive brain‐injured patients, showing that the internal mental experience of some patients was similar to that of healthy controls.

**Methods:**

In this study, patients with mild‐moderate AD (n = 9) and age‐matched healthy controls (n = 29) underwent a 40‐minute functional near‐infrared spectroscopy (fNIRS) scan. During the scan, participants watched two short movies, each with an intact‐ and scrambled‐plot version. Scrambled versions were included to demonstrate that synchrony was associated with higher‐order processing rather than the mere presentation of audio‐visual stimuli. Inter‐subject correlations (i.e., Pearson correlation coefficients between the hemodynamic activity of one or more AD patients and the remaining participants, including healthy controls) were used as the metric for synchrony.

**Results:**

Compared to the scrambled plot conditions, healthy controls showed robust synchronization in the frontal and parietal regions in the intact‐plot conditions. AD patients, in contrast, did not demonstrate a consistent pattern of synchronization in these regions during the intact‐plot conditions. Single‐subject analyses (i.e., comparing individual patients to the control group) further revealed that only one AD patient exhibited some degree of synchronization with the healthy controls during the audio‐visual stimuli.

**Conclusion:**

These preliminary findings indicate that AD patients may be experiencing the world differently from healthy individuals. The variability noted in synchronization across AD patients may be reflective of the heterogeneity in disease‐related impairments. Understanding deficits and patient needs from the lens of impaired conscious processing could support disease prognosis and promote improvements in person‐centered care.

